# Experimental Study on Fracture Behavior of Adhesive-Bonded Structure with V-notch Based on Digital Gradient Sensing Method

**DOI:** 10.3390/polym16142011

**Published:** 2024-07-13

**Authors:** Hai Yu, Yangzhuang An, Yunpeng Liu

**Affiliations:** 1 School of Civil Engineering, North Minzu University, Yinchuan 750021, China; 2 School of Materials Science and Engineering, University of Science and Technology Beijing, Beijing 100083, China

**Keywords:** adhesive-bonded structure, digital gradient sensing, angular deflection, fracture behavior, stress intensity factor

## Abstract

In this paper, a comparative study of the mode-I fracture behaviors of two types of specimens with a V-notch defect under plane stress conditions was performed using the digital gradient sensing (DGS) method. First, two types of specimens (namely one-piece specimen and bonded specimen) with the same V-notch defect were both made of polymethyl methacrylate (PMMA), and three different V-notch angles’ defect were considered for each type of specimen. Then, three-point bending tests were performed on both types of specimens. The angular deflection field of light near the V-notch region was recorded using a CCD during the experiments. Finally, by utilizing the relationship between the stress gradient and angular deflection as established by the elasto-optic effect, in conjunction with the principles of linear elastic fracture mechanics theory, the stress intensity factors (SIFs) of two types of specimens under different stress conditions were calculated using the least square method. According to the experimental results, the influence of V-notch angle on fracture load and fracture toughness of two kinds of specimens was discussed. Meanwhile, the experimental results show the significant differences in the fracture behaviors of the two types of specimens under mode-I loading conditions.

## 1. Introduction

In recent years, there has been an increasing demand for energy efficiency and environmental protection, which has led to the widespread adoption of lightweight design in various industrial sectors such as aviation, aerospace, automotive, and other industrial fields [1,2,3,4]. To achieve lightweight design and maximize material utilization, automobiles and aircraft are increasingly using advanced lightweight materials such as aluminum, magnesium, plastics, and composites. As a result, the proportion of steel used has decreased over the years, while the use of aluminum, non-ferrous metals, plastics, transparent plexiglass, rubber, and other lightweight materials has increased. For structures composed of the same or different types of lightweight materials, mechanical connection methods usually lead to stress concentration, local deformation, and other problems [5,6,7,8,9].

Furthermore, mechanical connection methods are inadequate for joining dissimilar or non-metallic materials due to the inherent properties (chemical, physical) of the materials involved. With the birth of a polymer structural adhesive, it brings new vitality to the unique connection bonding technology. Bonding technology is regarded as an ideal lightweight bonding technology because it can avoid the bottlenecks that occur in mechanical joining between dissimilar materials or non-metallic materials [10]. In practical engineering applications, it is an ideal choice to use a combination of bonding or composite connection (adhesive–mechanical connection) [11], which can compensate for the above defects. In addition, the application field of bonding technology is expanding with other advantages.

In industrial production, adhesive structures often take the form of adhesive joints [12]. The presence of stress singularity near the interface end of adhesive joints can lead to failures from static or fatigue loads starting at the interface end point, resulting in an instantaneous fracture [13,14]. This presents a significant safety concern. Therefore, it is crucial to study the stress singularity near the interface end and strength of the adhesive-bonded joint. The mechanical properties and mechanical behavior of adhesive joints have attracted the attention and research of many scholars. Single-lap joints made of aluminum and carbon fiber adherends were tested by Stuparu et al. [15] to understand better the behavior of such dissimilar joints. Local deformation fields were monitored by using the digital image correlation method (DIC). Wang et al. [16] revealed the interface behavior of iron-based shape memory alloy (Fe-SMA) strips bonded to metallic substrates to ensure the integrity of such joints. A series of single-lap shear tests was performed on Fe-SMA-to-steel adhesively bonded joints by using the DIC method. F. Heidarpour et al. [17] conducted an experimental investigation on the influences of the size and shape of 2D and 3D defects on the ultimate shear strength of the adhesive single-lap joints. The study concluded that there is an approximately linear decrease in the joint strength as the defect area increases in the case of the single-lap joints with 3D defects. However, when 2D defects are applied in an adhesive joint, a non-linear decrease in the joint strength is observed. He et al. [18] explored the failure mechanisms of a CFRP/aluminum adhesive joint, in which the DIC technique was used to record the fatigue process in real time. The literature above primarily investigated the mechanical properties and behaviors of adhesive-bonded joints through experimental methods. It is evident that digital image correlation [19,20], an advanced photomechanics technique, is increasingly utilized in the study of bonded joints due to the continuous development of computer and digital image processing technology. Because of the advantages of a simple optical setup, good environmental adaptability, wide measurement range, and high degree of automation, DIC has demonstrated compelling features for studying adhesive-bonded joints recently.

Transparent materials have a wide range of applications in modern industries and daily life, including optics, electronics, construction, automotive manufacturing, aerospace, and other fields. Adhesive connections between transparent materials or between transparent and other non-transparent materials play an important role in enabling the assembly and repair of products. In addition, the efficient use of transparent materials can be achieved through adhesive bonding technology. The bonding of transparent materials typically requires high strength, transparency, resistance to environmental corrosion, excellent optical properties, and reliable long-term stability. However, the current study of the fracture behaviors of bonded joints of transparent materials faces a number of challenges. Transparent materials are typically harder and more brittle, and their fracture behaviors differ from that of conventional materials. For instance, they have lower fracture toughness and are more prone to crack extension and fracture failure. Additionally, the microstructure and interfacial properties of transparent materials are crucial in determining their fracture behaviors, but are difficult to observe and analyze quantitatively due to their unique properties. Furthermore, the fracture behaviors of adhesive joints composed of transparent materials are influenced by several factors, including temperature, humidity, loading conditions, and environmental media. The complexity of these factors makes the study more challenging. Scholars have investigated the fracture problem of transparent materials using modern photomechanical methods. Digital gradient sensing (DGS) is a contemporary non-destructive testing technology that combines optical measurement [21,22], image processing, and a stress analysis. Based on the theory of photoelasticity, the method extracts stress information, especially the stress gradient, from the obtained strain field data, which can be used to evaluate the local stress concentration area of transparent materials and predict the potential fracture behaviors.

Dai et al. [23] proposed a compact stress measurement system for transparent objects based on the combination of photoelasticity and DGS methods. The interferometry fringe and speckle image of the deformed specimen were captured from perpendicular and slant directions, respectively. Yuan et al. [24] studied the stress field at the V-notch tip of polymer materials by using DGS methods. The results showed that the stress intensity factors at the V-notches extracted from the angular deflections agree well with the results calculated from the finite element method. Liu et al. [25] determined the local stress fields at the blunt V-notch tip by extending the DGS methods. Completely analytical expressions for angular deflections of light beams in the vicinity of the blunt V-notch tip were deduced based on Filippi’s stress equation.

While the DGS method has been applied to the stress concentration problem in transparent materials, its application to the adhesive structure of transparent materials was rarely studied, especially for the stress concentration problem and singular stress field when there are defects in the adhesive structure. In this paper, three-point bending experiments are performed on nonbonded and bonded structures with V-notch defects to investigate the fracture behaviors of the two types of specimens under mode-I loading. The effect of the V-notch defect size on the fracture behaviors of the two types of specimens is discussed, as well as the differences between the fracture behaviors of the two types of specimens.

## 2. Basic Principles and Methods

### 2.1. Basic Principles of DGS Method

**Figure 1:**
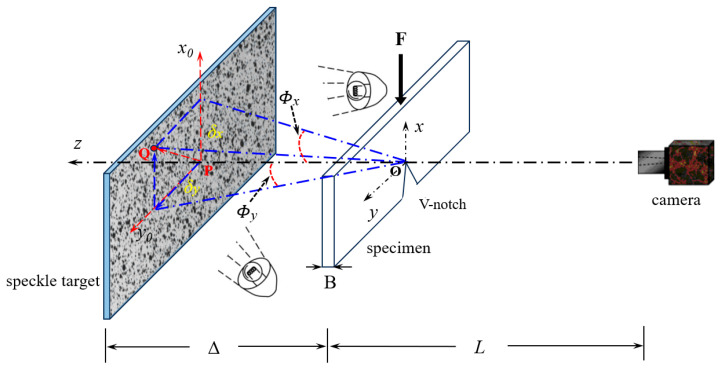
Schematic of basic principle and experimental setup for digital gradient sensing (DGS) method to determine planar stress gradients near V-notch tip.

The digital gradient sensing method was proposed by Periasamy and Tippur in 2012 [22]. This non-contact, full-field optical measurement technique is based on digital image correlation. The method establishes the relationship between the in-plane stress gradient and small deflection of light rays, using the elasto-optic effect exhibited by transparent materials due to the deflection of light rays caused by stress. The deflection angle of light rays can be obtained using the digital image correlation method. This method is sensitive to the in-plane stress gradient, making it suitable for investigating stress concentration problems such as a fracture under planar stress states. The working principle of the method and the governing equations are presented in Figure 1 and Equations (1) and (2), respectively.

(1) $$\phi_{x}\approx \frac{\delta_{x}}{\Delta }\approx \alpha_{x}=C_{\sigma }B\frac{\partial \left(\sigma_{x}+\sigma_{y}\right)}{\partial x}\;$$

(2) $$\phi_{y}\approx \frac{\delta_{y}}{\Delta }\approx \alpha_{y}=C_{\sigma }B\frac{\partial \left(\sigma_{x}+\sigma_{y}\right)}{\partial y}$$

In Equations (1) and (2), $\phi_{x}\;$ and $\phi_{y}\;$ are small deflection angles of light rays, B is the thickness of the specimen, $C_{\sigma }\;$ is the elasto-optic constant of the specimen ($-0.9\times 10^{-10}\;m^{2}/N$ for PMMA [21]), Δ is the distance between the specimen and target planes, and $\alpha_{x}$ and $\alpha_{y}$, which are related to in-plane stress gradients, are the direction cosines of the optical path due to the changes in the thickness and refractive index. Stress gradients in the *x*- and *y*-directions can be obtained by measuring the local displacement $\delta_{x}\;$ and $\delta_{y}$, which can be calculated by using two-dimensional digital image correlation between speckle images captured from the reference and deformed states.

### 2.2. Elastic Stress Field around the V-notch Tip

The linear elastic stress field for a V-notch plate subjected to an arbitrary in-plane loading can be expressed as the Williams series expansions [26,27]:

$$\left.\left\{\begin{array}{c} \sigma_{x}\\ \sigma_{y}\\ \tau_{xy} \end{array}\right.\right\}=\sum_{n=1}^{\infty }Re\left\{\frac{\lambda_{n}^{l}A_{n}}{r^{1-\lambda_{n}^{I}}}\left\{\begin{array}{c} \left(2+\lambda_{n}^{l}\cos2\alpha +\cos2\alpha \lambda_{n}^{l}\right)\cos\left(\lambda_{n}^{l}-1\right)\theta -\left(\lambda_{n}^{l}-1\right)\cos\left(\lambda_{n}^{l}-3\right)\theta \\ \left(2-\lambda_{n}^{l}\cos2\alpha -\cos2\alpha \lambda_{n}^{l}\right)\cos\left(\lambda_{n}^{l}-1\right)\theta +\left(\lambda_{n}^{l}-1\right)\cos\left(\lambda_{n}^{l}-3\right)\theta \\ -\left(\lambda_{n}^{l}\cos2\alpha +\cos2\alpha \lambda_{n}^{l}\right)\sin\left(\lambda_{n}^{l}-1\right)\theta +\left(\lambda_{n}^{l}-1\right)\sin\left(\lambda_{n}^{l}-3\right)\theta \end{array}\right\}\right\}$$

(3) $$+\sum_{n=1}^{\infty }Re\left\{\frac{\lambda_{n}^{\mathrm{II}}B_{n}}{r^{1-\lambda_{n}^{\mathrm{II}}}}\left\{\begin{array}{c} -\left(2+\lambda_{n}^{\mathrm{II}}\cos2\alpha -\cos2\alpha \lambda_{n}^{\mathrm{II}}\right)\sin\left(\lambda_{n}^{\mathrm{II}}-1\right)\theta +\left(\lambda_{n}^{\mathrm{II}}-1\right)\sin\left(\lambda_{n}^{\mathrm{II}}-3\right)\theta \\ \left(-2+\lambda_{n}^{\mathrm{II}}\cos2\alpha -\cos2\alpha \lambda_{n}^{\mathrm{II}}\right)\sin\left(\lambda_{n}^{\mathrm{II}}-1\right)\theta -\left(\lambda_{n}^{\mathrm{II}}-1\right)\sin\left(\lambda_{n}^{\mathrm{II}}-3\right)\theta \\ -\left(\lambda_{n}^{\mathrm{II}}\cos2\alpha -\cos2\alpha \lambda_{n}^{\mathrm{II}}\right)\cos\left(\lambda_{n}^{\mathrm{II}}-1\right)\theta +\left(\lambda_{n}^{\mathrm{II}}-1\right)\cos\left(\lambda_{n}^{\mathrm{II}}-3\right)\theta \end{array}\right\}\right\}$$

For the mode-I loading condition (or mode-I cracks), it can be simplified as follows:

(4) σxσyτxy=∑n=1∞ReλnAnr1−λn2+λncos⁡2α+cos⁡2αλncos⁡λn−1θ−λn−1cos⁡λn−3θ2−λncos⁡2α−cos⁡2αλncos⁡λn−1θ+λn−1cos⁡λn−3θ−λncos⁡2α+cos⁡2αλnsin⁡λn−1θ+λn−1sin⁡λn−3θ  

**Figure 2:**
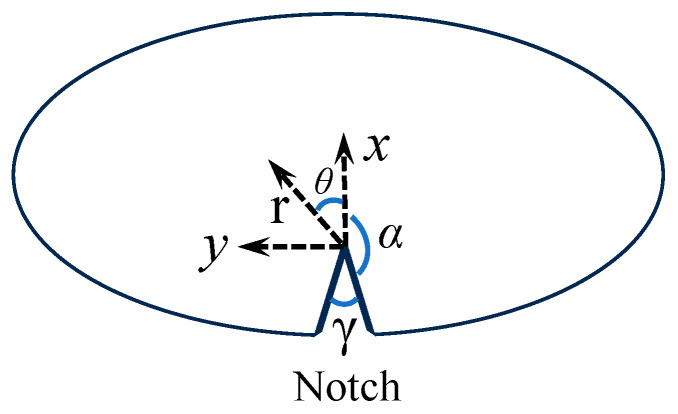
Schematic of V-notch and Cartesian coordinate system.

In Equation (4), $\left(r,\theta \right)\;$ are the polar coordinates as shown in Figure 2, *n* is the order of term in the infinite series, Re () denotes the real part of (), *γ* is the notch angle and its relationship with angle α can be expressed by the following equation: $\alpha =\frac{2\pi -\gamma }{2}$, and $\lambda_{n}$ is the order of singularity, which is dependent on the V-notch angle and can be expressed as

(5) $$\lambda_{n}\sin2\alpha +\sin2\lambda_{n}\alpha =0$$

The above equation can be solved by the numerical iteration method to obtain the specific value of $\lambda_{n}$ at each V-notch angle. In Equation (4), the terms involving coefficients $A_{n}$ correspond to the mode-I expansions. The first term (n = 1) is singular and its coefficient $A_{1}$ is related to the mode-I notch stress intensity factor $K_{I}^{V}$ defined as

(6) $$K_{I}^{V}=\underset{r\to 0}{lim}\left(\sqrt{2\pi }r^{1-\lambda }\sigma_{\theta }\left(\theta =0\right)\right)=\sqrt{2\pi }\lambda \left(1+\lambda -\lambda \cos2\alpha -\cos2\alpha \lambda \right)A_{1}$$

The values of λ for the different notch angle γ are summarized in Table 1.

### 2.3. Solving Equation of $K_{I}^{V}$

Extracting the stress intensity factor $K_{I}^{V}$ of the V-notch tip from the deflection angle field obtained by the DGS measurement method, it is necessary to derive the solution equation that contains the stress intensity factor as an unknown. From Williams’ asymptotic stress field expansion for mode-I cracks, the stress gradient in the *x*-direction and *y*-direction can be expressed as

(7) $$\frac{\partial \left(\sigma_{x}+\sigma_{y}\right)}{\partial_{x}}=4\sum_{n=1}^{\infty }Re\left\{\left(\lambda_{n}-1\right)\lambda_{n}A_{n}r^{\lambda_{n}-2}\left(\cos\left(\lambda_{n}-2\right)\theta \right)\right\}$$

(8) $$\frac{\partial \left(\sigma_{x}+\sigma_{y}\right)}{\partial_{y}}=4\sum_{n=1}^{\infty }Re\left\{\left(\lambda_{n}-1\right)\lambda_{n}A_{n}r^{\lambda_{n}-2}\left(\sin\left(\lambda_{n}-2\right)\theta \right)\right\}$$

If K-dominance is assumed (or, terms corresponding to N ≥ 2 in Equations (7) and (8) are neglected), combining Equations (1) and (2), we can obtain

(9) $$\phi_{x}=C_{\sigma }B\frac{\partial \left(\sigma_{x}+\sigma_{y}\right)}{\partial x}\approx 4C_{\sigma }B\left(\lambda_{1}-1\right)\lambda_{1}A_{1}r^{\lambda_{1}-2}\left(\cos\left(\left(\lambda_{1}-2\right)\theta \right)\right)$$

(10) $$\;\;\;\phi_{y}=C_{\sigma }B\frac{\partial \left(\sigma_{x}+\sigma_{y}\right)}{\partial x}\approx 4C_{\sigma }B\left(\lambda_{1}-1\right)\lambda_{1}A_{1}r^{\lambda_{1}-2}\left(\sin\left(\left(\lambda_{1}-2\right)\theta \right)\right)$$

Using the above solving equations, the mode-I stress intensity factor can be obtained by performing an over-deterministic regression analysis of the measured data.

## 3. Experiment Process

### 3.1. Specimen Preparation

**Figure 3:**
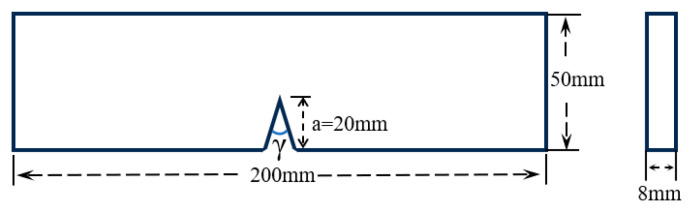
The geometry of the specimens.

**Figure 4:**
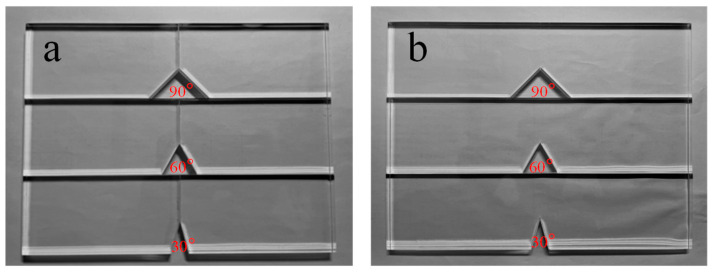
Physical pictures of two types of specimens: (**a**) Bonded specimens, (**b**) One-piece specimens.

Comparative experiments in this study utilized both bonded and one-piece specimens. Both types of specimens had the same geometric dimensions and V-shaped notches (see Figure 3), and were made from transparent polymethyl methacrylate (PMMA). The material properties and stress optical constant of the PMMA specimens can be found in Table 2. Additionally, this study employed the same three V-notch angles (30°, 60°, and 90°) for both types of specimens. The bonded specimens were joined using a UV shadowless specific adhesive designed for PMMA (Shenzhen KSIMI K2018, Shenzhen, China). Before bonding, the surfaces were polished with 400-mesh waterproof sandpaper to ensure flatness of the bonding surface, then cleaned with acetone. The adhesive was evenly applied for seamless butt joint bonding, and the specimens were aligned precisely before exposure to ultraviolet light for 30 s. Following this, the specimens were left at room temperature for 24 h. Detailed physical illustrations of the two specimen types can be found in Figure 4.

### 3.2. Experimental Setup

**Figure 5:**
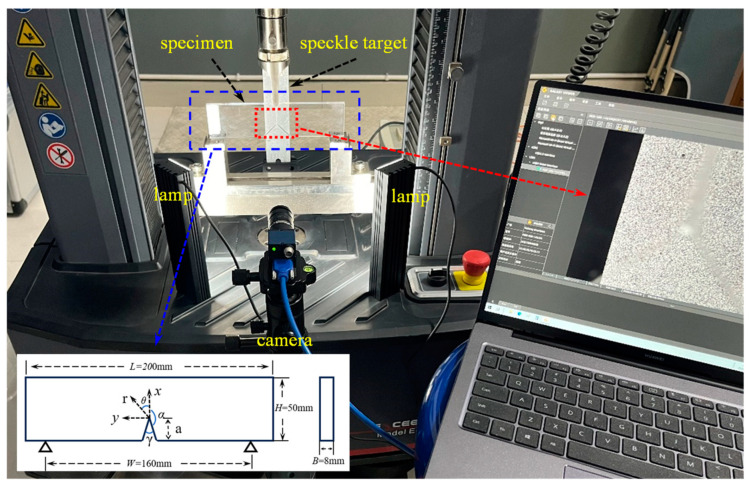
Experimental setup used to measure angular deflections of light rays caused by mode-I quasi-static loading.

Figure 5 shows the experimental setup used to measure the angular deflections of light resulting from a V-notch in planar three-point bending PMMA specimens under quasi-static loading. The setup includes an electronic universal testing machine (E44.304 MTS), a CCD camera (MER2-630-60U3M, Daheng Image Co., Ltd., Beijing, China), a speckle target with randomly and uniformly distributed speckle spots, a transparent PMMA specimen with a V-notch, and two illumination sources. The speckle target was a planar surface, which was painted with random uniform black and white spots and was placed at a distance of Δ = 160 mm behind the mid-plane of the specimen. The transparent specimen was rested on two anvils (span *W* = 160 mm) and loaded by a loading head via the universal testing machine placed in front of the target plane. The CCD camera with 3088 × 2064 black and white pixels was fitted with a lens with a focal length of 35 mm, and positioned in front of the specimen at a sufficient distance of 550 mm. During the experiment, the speckle target needs to be assisted by light. To obtain a uniform intensity gray level, symmetrical double light sources were used. By adjusting the intensity of the light source and fine-tuning the focal length, it is ensured that the camera will acquire images with an appropriate grayscale and sharpness. In the displacement control mode, a slow loading rate of 0.2 mm/min is performed.

**Figure 6:**
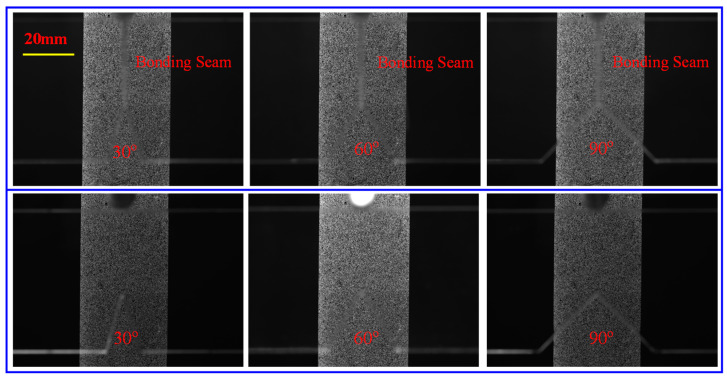
Speckle images near the V-notch of the two types of specimens taken using the camera before loading (row 1 is bonded specimens and row 2 is one-piece specimens).

Before loading, one image was captured as the reference image (shown in Figure 6). Subsequently, images were captured every 25 *N* as the sequence target images during the loading process. As the V-notch vicinity suffered deformation, light rays passing through the specimen were deflected by the local non-uniform state of stress distorting the speckle images relative to the reference state, and this distorted deformation is especially obvious at the V-notch. Therefore, to ensure that the actual deformation matches the shape function adopted by the algorithm as much as possible, a relatively small computational subset of 31 pixel × 31 pixel was adopted for the correlation operation using the 2D DIC.

## 4. Results and Discussion

### 4.1. Analysis of Angular Deflection Fields

**Figure 7:**
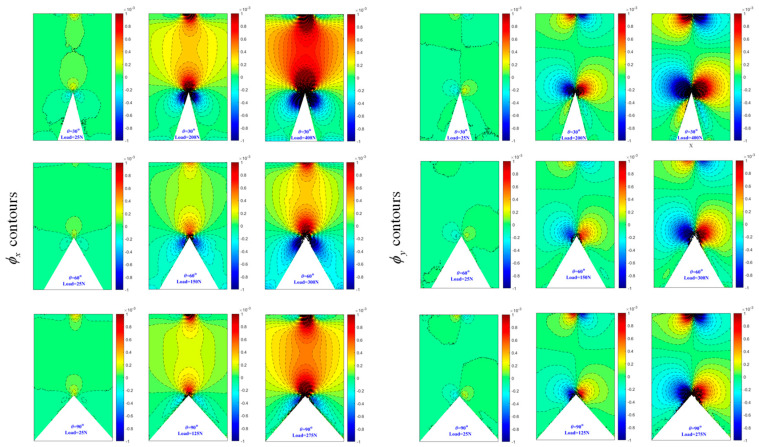
Experimentally determined angular deflection contours [$\phi_{x\;}$ (**left**) and $\phi_{y}$ (**right**)] of one-piece specimen near V-notch for different load levels. Contour interval is $5\times 10^{-5}$ radian.

**Figure 8:**
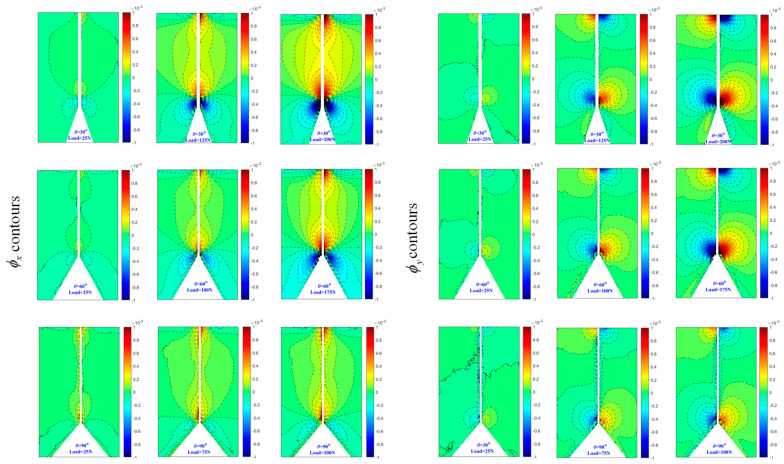
Experimentally determined angular deflection contours [$\phi_{x\;}$ (**left**) and $\phi_{y}$ (**right**)] of bonded specimen near V-notch for different load levels. Contour interval is $5\times 10^{-5}$ radian.

Figure 7 and Figure 8 display the contours of $\phi_{x}$ and $\phi_{y}$ for three load levels under each V-notch angle within a rectangular region surrounding the V-notch. The $\phi_{x}$ contours exhibit symmetry about the *x*-axis, while the $\phi_{y}$ contours show antisymmetry about the *x*-axis. As the load level increases, the contour lines become denser, particularly near the V-notch area, indicating a steep stress gradient at the V-notch. It is important to note that the contour lines near the narrow band area adjacent to the bonding surface in Figure 8 appear blurred due to the presence of a zero-thickness adhesive layer. Furthermore, the contours appearing at the top of all subplots in Figure 7 and Figure 8 are a result of the indenter’s action, causing light deflection in the surrounding region. Likewise, the contours of $\phi_{x}$ and $\phi_{y}$ around the indenter region exhibit symmetry and antisymmetry about the *x*-axis, respectively.

### 4.2. Extraction of SIFs

According to Equations (9) and (10), the mode-I SIFs can be determined after calculating the angular deflection fields using the 2D DIC method. For the two types of specimens discussed in this paper, identical calculation regions are used to determine the SIFs. Specifically, SIFs for the two types of specimens are calculated using the region of 0.3 ≤ *r/B* ≤ 1.2 and an angular extent, −120° ≤ θ ≤ 120°. The SIFs are extracted by least squares from the data points contained in the above regions, which can effectively reduce the influence of the data points too far or too close to the V-notch on the calculation results. It should be noted that to ensure accurate calculation results for a bonded specimen, it is important to avoid the influence of invalid data in the vicinity of the bonding surface on the calculation results.

**Figure 9:**
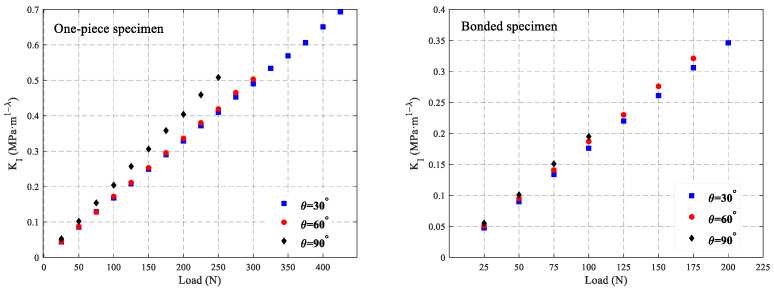
Experimentally determined stress intensity factors from measured angular deflections near a mode-I static three-point bending V-notch defect problem for different applied load levels.

Equations (9) and (10) can be used to calculate the SIFs under different loads using the deflection angles $\phi_{x}$ and $\phi_{y}$, respectively. This study employs the average value of $K_{Ix}$ and $K_{Iy}$ to represent the SIFs $K_{I}$, i.e., $K_{I}=\frac{{\left(K\right)}_{Ix}+K_{Iy}}{2}$, where $K_{Ix}$ is the SIFs determined using $\phi_{x}$ and $K_{Ix}$ is the SIFs determined using $\phi_{y}$. Figure 9 shows the SIFs of the two types of specimens under different loads. It demonstrates that SIFs of the two types of specimens under different V-notch angles are basically linear with the load, and the SIFs increase linearly with the increase in the load. Additionally, the V-notch angle has a certain influence on the SIFs. Under the same load condition in the experiments conducted for the three angles selected in this study, the SIFs increase with the increase in the notch angle, and the extent of this increase is related to the magnitude of the load. However, under the same load and identical V-notch angle, the SIFs for the two types of specimens are essentially consistent, showing no significant differences. As can be seen in Figure 9, for bonded specimens, the effect of the size of the V-notch angle on the stress intensity factor is not obvious, but it can still be seen that a large V-notch angle corresponds to a relatively large stress intensity factor under the same load. However, for the one-piece specimen, when the V-notch angle is 90 degrees, the stress intensity factor under the same loading condition is significantly higher than that under the other two angles, which indicates that the effect of the size of the V-notch angle on the stress intensity factor of the one-piece specimen is significantly larger than that on the stress intensity factor of the bonded specimen.

**Figure 10:**
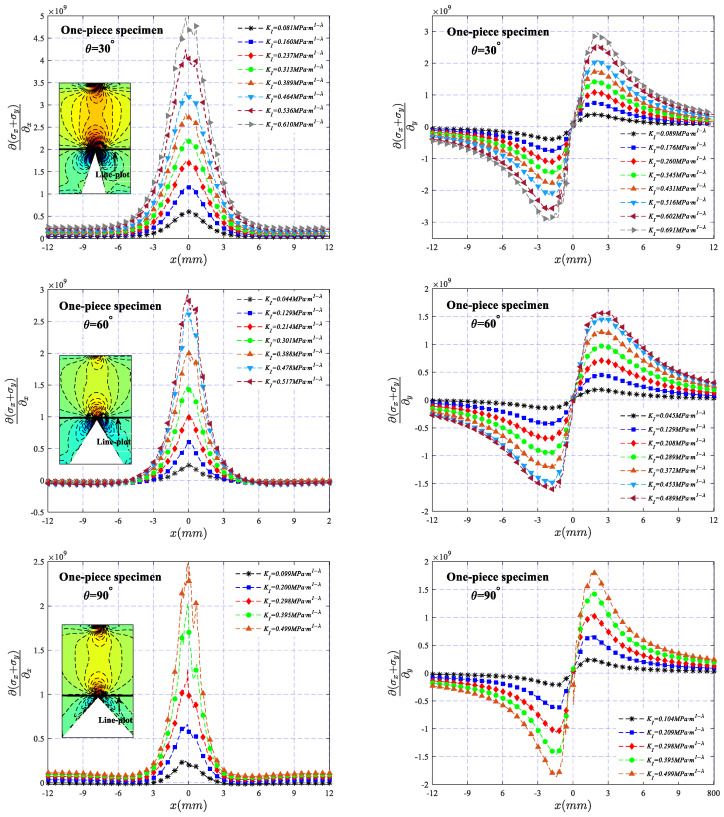
The variation of $\frac{\partial \left(\sigma_{x}+\sigma_{y}\right)}{\partial x}$ and $\frac{\partial \left(\sigma_{x}+\sigma_{y}\right)}{\partial y}$ along the net section of the one-piece specimen across a line at a vertical distance of 1 mm from the tip of the V-notch.

**Figure 11:**
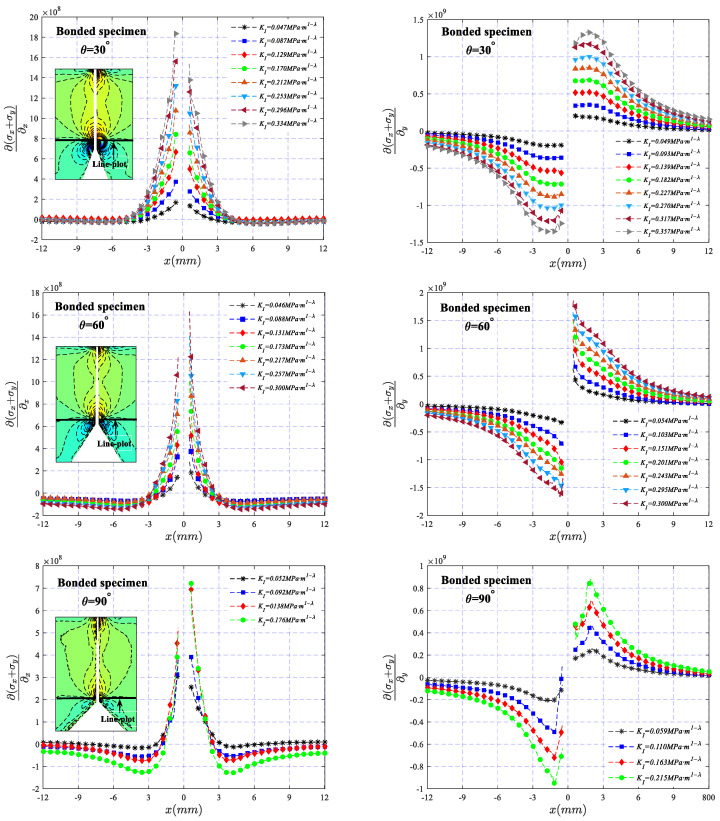
The variation of $\frac{\partial \left(\sigma_{x}+\sigma_{y}\right)}{\partial x}$ and $\frac{\partial \left(\sigma_{x}+\sigma_{y}\right)}{\partial y}$ along the net section of the bonded specimen across a line at a vertical distance of 1 mm from the tip of the V-notch.

Under different SIF values, the variation of $\frac{\partial \left(\sigma_{x}+\sigma_{y}\right)}{\partial x}$ and $\frac{\partial \left(\sigma_{x}+\sigma_{y}\right)}{\partial y}$ (calculated by Formulas (1) and (2)) along the net section of the two types of specimens across a line at a vertical distance of 1 mm from the tip of the V-notch is illustrated in Figure 10 and Figure 11, respectively. It can be observed that the stress gradient exhibits a symmetric and antisymmetric distribution about the *x*-axis, and the value increases with the increase in *K_I_*, indicating that the degree of stress concentration becomes more apparent as the load increases. In addition, for the two types of specimens, there is a slight difference in the stress distribution in the region near the V-notch. The high-stress area of the bonded specimen is mainly distributed in the band area where the bonding layer is located, while the high-stress area of the one-piece specimen is obviously larger than that of the bonded specimen, making the stress dominant region of the bonded specimen smaller than that of the one-piece specimen, which may be one of the main reasons that the fracture toughness of the bonded specimen is obviously smaller than that of the one-piece specimen. It should also be noted that the absence of data in the region of the adhesive layer in Figure 11 is due to the presence of the adhesive layer, making the image of the region blurred, resulting in an inability to perform an accurate calculation of the deflection angle.

### 4.3. Fracture Toughness

**Figure 12:**
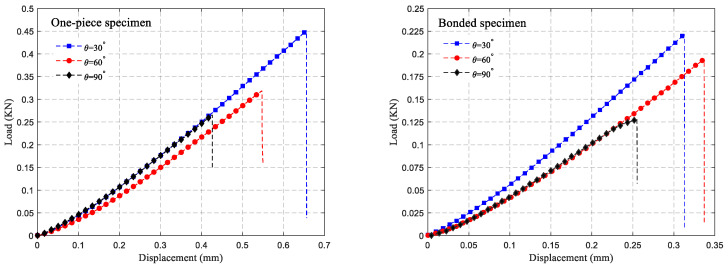
Displacement–load curves of two types of specimens under different V-notch angles.

**Figure 13:**
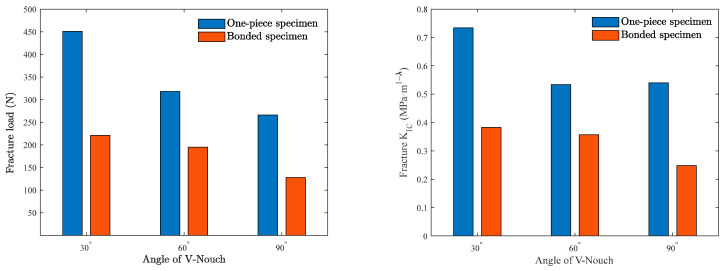
Fracture load and fracture toughness of two types of specimens under different V-notch angles.

The force–displacement curves of the two types of specimens during the loading process are illustrated in Figure 12. It is clear that both types of specimens exhibit a linear relationship between force and displacement during loading, with a brittle fracture occurring at the end of loading. It can also be seen from Figure 12 that the fracture loads of both types of specimens are closely related to the size of the V-notch, with larger V-notch angles corresponding to smaller fracture loads. The one-piece specimens fractured vertically along the V-notch, whereas bonded specimens fractured along the adhesive surface at the V-notch. The fracture loads of specimens were precisely recorded by the force sensor of the universal testing machine. Based on Figure 9, the relationship between the stress intensity factor and load suggests that the fracture toughness K_IC_ of each sample can be determined through linear fitting. As the V-notch angle increased, the fracture loads of both types of specimens decreased gradually, with bonded specimens showing a notably lower fracture load compared to one-piece specimens. The fracture loads of bonded specimens were 49%, 61%, and 48% of the fracture load of one-piece specimens under the three different V-notch angles, respectively. Moreover, the fracture toughness of the two types of specimens does not decrease entirely with the increase in the V-notch angle, as shown in Figure 13. It is evident from Figure 13 that the fracture toughness of the one-piece specimen at a 90° V-notch angle slightly increases compared to the 60° V-notch angle. In contrast, the fracture toughness of the bonded specimen decreases gradually as the V-notch angle increases. Specifically, under three different V-notch angles, the fracture toughness of the bonded specimen is 52%, 67%, and 46% of the fracture toughness of a one-piece specimen.

## 5. Conclusions

This paper presents a comparative study of the mode-I fracture behaviors and stress concentration of PMMA bonded and non-bonded specimens with different V-notch angles under plane stress. This study employs full-field optical measurement (DGS) to extract the stress intensity factors of the V-notch angles under different stress states. The stress intensity factors of the V-notch angles under different stress states are extracted by three-point bending experiments to characterize the differences in the fracture behaviors of the two types of specimens. Some important conclusions that are in this article are as follows:

- By carrying out DGS experiments on PMMA bonded and one-piece specimens with different V-notch defects, the stress field near the V-notch of the two types of specimens under the plane stress state was characterized, and the fracture behaviors of the two types of specimens were compared. The effectiveness of the DGS method in the study of the stress concentration of two types of specimens under plane stress is proved.
- In this study, the adhesive used resulted in a lower fracture load for bonded specimens compared to one-piece specimens in three-point bending tests. The fracture loads of bonded specimens were 49%, 61%, and 48% of the fracture load of one-piece specimens at three different V-notch angles. Additionally, the fracture loads of both types of specimens decreased as the V-notch angle increased.
- The fracture toughness of the two types of specimens in the three-point bending test showed significant differences. The fracture toughness of the bonded specimen was 52%, 67%, and 46% of that of the one-piece specimen at the three V-notch angles, respectively. The fracture toughness of both types of specimens was found to be dependent on the size of the V-notch angle, with a more pronounced effect observed in the bonded specimen. This variation highlights the distinct fracture behaviors exhibited by the two types of specimens.

## Figures and Tables

**Table 1:** The value of λ for different notch angle *γ*.

*γ*	30°	60°	90°
α	165°	150°	135°
λ	0.5014	0.5123	0.5445

**Table 2:** The material properties of the PMMA specimen.

Material	PMMA
Elastic modulus E(GPa)	3.33
Poisson’s ratio υ	0.35
Stress optical constants ${\;C}_{\sigma }$	$-0.9\times 10^{-10}$

## Data Availability

Data are contained within the article.

## Abbreviations

The following abbreviations are used in this manuscript:

DIC	Digital image correlation
DGS	Digital gradient sensing
SIFs	Stress intensity factors
